# 

**Published:** 1895-09-07

**Authors:** 


CONTENTS.

**The Attractions of Medicine** - 387

**Clocks and Heart Disease** - 388

**On Modern Progress in Ophthalmic Medicine and Surgery.** By R. B. Carter, F.R.C.S.—Diseases of the Cornea (*continued*) - 389

**The Commencement of Medical Study** - 393

**Annotations.** — London Must Give Degrees — London Wants Students—The Doctor on Wheels - 391

**Notes and News** - 392

**The Medical Schools of London** - 395

**Medical Progress and Hospital Clinics—**

Progress in Surgery.—Orthopædic Surgery - 393

New Appliances and Things Medical - 394

**The Institutional Workshop—**

Within the Hospitals.—The Canton Hospital at Geneva - 401

The Story of the Insane from Year to Year - 401

Practical Departments - 402

Visit of the Shahzada to the St. Thomas's Hospital - 402

**Editor's Letter-box** —The Throat Hospital, Golden Square - 403

**Scraps and Gleanings** - 402

**The Student of Medicine** - 403

EXTRA SUPPLEMENT.—THE NURSING MIRROR.

**News from the Nursing World.**—The Home or the Workhouse—A Story in a Tea-pot—How Small-pox Patients are Nursed—A Guardian's Testimony—A Holiday in Bed—A Hard-hearted Nurse.—Peeping Tom!—Home Nursing in Stockport—A Curious Question - cliii

**Wanted—Nurses** - clv

**Reminiscences of "First Aid"** - clvi

**Elementary Physiology for Nurses.** By C. F. Marshall, M.D., B.Sc., F.R.C.S. V.—The Circulatory System (*continued*) - clv

**Nursing in Rome.** I.—Santo Spirito Hospital on the Tiber clvi

**Novelties for Nurses** - clvii

**On the Choice of Pupils for Professional Nursing** - clvii

**"The Hospital" Convalescent Fund** - clviii

